# Transition to Practice: A Novel Life Skills Curriculum for Emergency Medicine Residents

**DOI:** 10.5811/westjem.2018.10.39868

**Published:** 2019-01-01

**Authors:** Holly Caretta-Weyer

**Affiliations:** Stanford University School of Medicine, Department of Emergency Medicine, Palo Alto, California

## BACKGROUND

Transitions are a familiar topic in medical education. Of particular interest to medical educators in recent years has been the need to ease the intense and stressful experience of transitioning from preclinical to clinical undergraduate medical education and from medical school to residency, while relatively little attention has been paid to examining the transition from residency to independent practice.^1^–^2^

The transition from residency to independent academic or community practice as an attending physician is a vulnerable time that presents significant challenges including final responsibility for patient care, management and leadership tasks, the education of residents, a new workplace environment and colleagues, and practice management skills.^3^ In addition to these workplace-based challenges, graduating residents often cite deficiencies in practical life and job skills such as preparing a curriculum vitae (CV) and cover letter, contract negotiation, personal finance, and time management.^3^–^4^ Many residency programs touch on some of these topics throughout each resident’s time in training; however, despite the gravity and generalizability of the subject matter, there is little published evidence of broad-based, fully-developed, evidence-based curricula in emergency medicine (EM) devoted to teaching senior residents to successfully navigate these issues while transitioning to independent practice.^4^–^7^

## GOAL OF CURRICULUM AND OBJECTIVES

We developed a multi-modal, learner-driven, interactive curriculum to address the unique nonclinical challenges senior EM residents face during the transition from residency to independent practice. The overarching goal of this curriculum is for residents to cultivate the necessary life skills in each of these domains to successfully navigate the transition to independent practice and beyond. Specific objectives were determined by the targeted needs assessment of the residents and junior faculty (Table 1).

## CURRICULAR DESIGN

We used the framework of Kern’s six-step model for curriculum development in medical education in developing this curriculum.^8^ A targeted needs assessment of current residents and junior faculty in both academic and community settings in our geographic area identified nine topics for inclusion in this pilot curriculum, one to be covered each month over the course of the curriculum in a just-in-time format. These topics are shown in Table 1.

The first session of the curriculum preceding the topic sessions is an interactive panel with recent graduates in academic positions, fellowships, and community practice to discuss how to obtain a position in each of these practice areas and answer questions. This is followed monthly by sessions that use learner-driven instructional methods including group processes such as team-based learning and role-playing, self-directed learning via reflection and learning plan development, and practical application of skills by developing artifacts and obtaining feedback for improved performance. Table 1 demonstrates the objectives, instructional design, and implementation strategies for each session.

We chose an interactive format as residents needed to produce tangible products and learn to use these skills as part of the curriculum. A largely learner-driven strategy was selected due to limitations in available resources. The most significant resource required for this curriculum is time. Faculty time is needed to review documents or role-play scenarios and provide feedback to the residents. We unfortunately lacked dedicated conference time for this content; thus, this curriculum was delivered outside of typical didactic time on various evenings at faculty homes or restaurants. If time were allotted during didactic conference for class-specific content, this would be an ideal curriculum for senior residents. Obtaining buy-in from program and departmental leadership to support this curriculum is crucial to its success.

## IMPACT/EFFECTIVENESS

To evaluate the outcomes of this pilot curriculum we used a program-oriented approach, focusing on the extent to which the curricular objectives were successfully delivered and achieved via the tangible outcomes associated with each session, which were observed in real time. The deliverables of each session were achieved as stated in the objectives, as determined by the curriculum director and the session faculty leaders. Additionally, we employed a participant-oriented evaluation approach using a mixed-methods, survey-based format, including quantitative questions regarding the importance of the content covered in the transition to independent practice, whether the objectives were met during the session, and how well residents felt prepared for each component of the transition to practice after participating. This was followed by an open-ended feedback section for descriptive comments regarding the benefits and areas for improvement of each session.

The quantitative evaluation survey questions employed a five-point Likert scale with the following anchors: 1=strongly disagree, 2=disagree, 3=neutral, 4=agree, and 5=strongly agree. Using Messick’s framework of validity,^18^ we addressed two areas of validity evidence in developing our evaluation survey questions. By developing the evaluation to match the content delivered in direct consultation with the content experts for each session, as well as receiving feedback on the questions from two medical education experts outside our department at our institution, this provided content validity. We piloted the survey on five second-year residents and three members of the residency education leadership team for clarity of the questions, relevance to the content covered, and grammatical errors. Edits were made based upon critiques received from the respondents, addressing response process validity.

The survey was administered to the residents in attendance at each session. There were eight residents at each workshop out of 11 members of the senior class (due to some covering clinical responsibilities). All eight residents in attendance at each session completed the survey. The results of this evaluation are presented in Table 2. Other key stakeholders including residency program leadership and core faculty who taught or provided feedback to the residents within the curriculum also provided valuable feedback regarding the curriculum that mirrored the resident responses.

This curriculum was piloted on 11 senior EM residents. Post-curriculum implementation surveys were analyzed and coded for themes by the author. During the evaluation phase, residents expressed greater confidence in the application, interview, and contract negotiation process for their first position after residency due to participating in the pilot of this curriculum. All stated that they felt this curriculum had prepared them to face the transition to independent practice and alleviated much of their anxiety. Additionally, they felt that they could apply many of these topics to their current practice in residency, specifically citing the billing and coding and time management sessions.

Residency program leadership evaluated the positive feedback from these sessions and is working to make them a regular component of the EM didactic curriculum. Additionally, our core faculty have expressed regret at not having received similar training when they were residents.

## LIMITATIONS

While there are few published curricula covering the transition to practice within EM, there are likely several programs covering some or potentially all of this content already. A national needs assessment and survey to identify what is currently being done across all programs may inform the literature further on this topic. Additionally, the conference and faculty time required to implement this curriculum proved onerous to our program during the pilot phase, requiring outside time for implementation. A significant investment on the part of the program and faculty for class-specific content during conference time and incentivization of the faculty to participate may be necessary to make this a successful endeavor at each program. Finally, selected comments were provided from the evaluations of each curricular session. These comments were reviewed and selected by the author, and while attempting to remain impartial and report comments that are representative of all those received, this may have resulted in selection bias. The reporting of the quantitative post-implementation evaluation data as well as the constructive feedback was provided in an attempt to ameliorate this potential for bias.

## CONCLUSION

This multi-modal, learner-driven, interactive curriculum was well received within our EM residency program. It could also be adapted to any graduate medical education training program with minor, specialty-specific adjustments given the wide applicability of these skills for residents in all specialties as they navigate the transition to independent practice. Going forward, it will be important to gather more objective outcomes in order to determine the ultimate value of this and other future curricular initiatives addressing the transition to practice.

## Figures and Tables

**Table 1:** Objectives, instructional methods, and implementation strategies for each session of the transitions-to-practice curriculum.

Session topic	Objective	Instructional design and implementation
Developing a CV and cover letter	Design a cover letter that includes a statement of intent, your unique qualifications, and how these qualifications fit with your target position. Prepare a CV with sufficient detail and appropriate sections based upon the position for which you are applying.	Artifact development and feedback^9^ - Residents review the cover letter and CV of recent graduates who were successful in obtaining a position in their desired practice environment. They then produce their own CV and cover letter and receive feedback from faculty on their work.
Interview strategies	Employ interview strategies to provide appropriate answers based upon question type and the job for which you are applying.	Role-playing^9^–^10^ - Faculty role-play interview questions with the residents based upon the practice setting they intend to enter.
Contract negotiation	Use key contract-negotiation strategies when discussing salary, benefits, shift count, new role, expectations, and other key aspects of your first contract after residency.	Role-playing^9^–^10^ - Residents review sample contracts within their target practice setting and market with a faculty member to review pearls and pitfalls. Residents then role-play with faculty how to negotiate various aspects of their contract.
Time management	Develop a system for task prioritization, time blocking, and saying yes or no to new opportunities. Apply time management strategies to maximize productivity and minimize distractors.	Group discussion and think-pair-share^9^–^10^ - Residents discuss time blocking and task prioritization systems and develop a Covey 2×2 table based upon their priorities. They then think-pair-share to identify ways in which to maximize their productivity to achieve their goals.
Burnout prevention	Analyze prospective difficulties in your first year of independent practice and how these may put you at risk for burnout.	Narrative medicine^11^–^13^ - Residents and faculty present stories of difficult cases and life situations and use reflective writing to process each other’s stories. This is followed by debriefing and discussing useful tools for mindfulness and burnout prevention.
Medicolegal pitfalls	Compare approaches to clinical cases that are at high risk for litigation in emergency medicine.	Team-based learning^9^,^14^–^15^ - Using real-life, de-identified cases that have led to litigation in the past, residents form teams to discuss and debate their approach to these scenarios. If no cases are available, there are books with several examples.
Personal finance management	Apply principles from the book The White Coat Investor to develop a personal budget for your first year out in independent practice.	Book club and budget preparation^9^ - Residents read The White Coat Investor prior to attending the session. They then discuss it in a book club format. Finally, they develop a personal budget based upon the book and their discussion.
Billing and coding	List the necessary elements from the history of present illness, review of systems, physical exam, and medical decision-making sections of a chart required to bill for each level (1–5).	Chart review^16^–^17^ - Residents review their own charts and those of their faculty and assign a level to each chart for billing purposes. They then compare their results to that of the medical coders and discuss the results and strategies for improvement as a group.

*CV*, curriculum vitae.

**Table 2:** Post-session evaluation questions with quantitative responses rated on a 1–5 point Likert scale (mean and standard deviation reported) and representative qualitative comments from the evaluation forms.

Session topic	This topic was very important for me to learn as part of my transition to practice (n=8) mean (SD)	The session organizers met the objective(s) for this session (n=8) mean (SD)	I feel prepared in this content area after attending this session n=8 mean (SD)	Please provide your feedback regarding the benefits of this session and suggestions for improvement
Developing a CV and cover letter	4.75 (0.46)	4.87 (0.35)	4.5 (0.53)	“It was extremely helpful to have faculty review my CV and cover letter before applying for fellowship.” “It would be useful to have more community partners review our CVs to tailor them more to what community hiring directors are looking for.”
Interview strategies	5 (0)	4.62 (0.52)	4.75 (0.46)	“This really takes the guesswork out of interviewing for jobs!” “It would be awesome to have more faculty, so we could have the opportunity to do more of these mock interviews kind of like when we do oral boards practice.”
Contract negotiation	5 (0)	4.87 (0.35)	4.62 (0.52)	“This is something I was so afraid of going into the job search - without this, I would have had no idea what to do!” “It would be helpful to see sample contracts from all of the groups in the area.”
Time management	4.5 (0.53)	4.87 (0.35)	4.62 (0.52)	“I thought I knew everything about time management, but this session gave me a whole new approach! I wish I would have learned this sooner in residency!” “It would be even better next time if we had a follow-up session to get feedback on how we are doing and what to adjust to get the most out of our system.”
Burnout prevention	4.87 (0.35)	5 (0)	4.75 (0.46)	“I went into this thinking we would be meditating (yuck) - sharing our stories amongst the faculty and residents and the camaraderie that was built really helped me realize what I need to do to take care of myself both personally and professionally well into the future.” “We should have more of these sessions on a regular basis!”
Medicolegal pitfalls	5 (0)	4.75 (0.46)	4.37 (0.52)	“This session was extremely eye opening - it really helped me realize how I need to approach every patient, every chart.” “This session was somewhat stressful - it would be great to have a debriefing and normalizing portion afterward.”
Personal finance management	5 (0)	4.87 (0.35)	4.62 (0.52)	“This is by far the most important thing I needed to know for my life prior to graduation!” “It would be helpful to have follow-up resources or contacts where we are going to be living/working in order to follow up on these principles.”
Billing and coding	4.87 (0.35)	5 (0)	4.87 (0.35)	“This session was really helpful for getting us ready to go out and practice in the real world since this is not something we think about as residents at all!” “I really wish we would have had this as interns, so I could have been charting like this all along!”

*CV*, curriculum vitae; *SD*, standard deviation.

*SD*, standard deviation.
